# The preface: Toward higher-*T*_c_ superconductivity under lower pressure—from binary to ternary superhydrides

**DOI:** 10.1093/nsr/nwae210

**Published:** 2024-07-03

**Authors:** Fu-Chun Zhang, Ho-Kwang Mao, Xin-Cheng Xie

**Affiliations:** Kavli Institute for Theoretical Sciences, University of Chinese Academy of Sciences, China; Shanghai Advanced Research in Physical Sciences, China; Center for High Pressure Science and Technology Advanced Research, China; International Center for Quantum Materials, School of Physics, Peking University, China

## Abstract

This is the Preface to Special Topic: Challenges to Achieving Room-Temperature Superconductivity in Superhydrides under Pressure.

Superconductivity—the phenomenon of complete vanishing of electrical resistance and expulsion of magnetic field—is the paramount electromagnetic property for technological advancement that is only shackled by the requirement for an extremely low superconducting critical temperature (*T*_c_). The discovery of a *T*_c_ of >200 K in hydrogen sulfide at 200 GPa of pressure [1] broke the 20-year high-*T*_c_ record of 164 K in mercury cuprate at 30 GPa, and stimulated tremendous interest in the scientific community. Intensive quests for higher-*T*_c_ records in hydrogen-dominant compounds have led to the discoveries of dozens of superconductive superhydrides approaching room-*T*_c_ under high pressures. *NSR* organized this Special Topic to assess the current status and future perspective of this exciting research field. As depicted by the cover picture, the Mt Everest metaphor for room-*T*_c_ superconductivity is now within reach.

The breakthrough is a classical example of the combined efforts of theory and experimentation. Metallic hydrogen under extremely high pressure was theoretically proposed by Neil Ashcroft [2] to be a high-*T*_c_ superconductor due to its light mass based on Bardeen–Cooper–Schrieffer theory. Ashcroft extended his conjecture to hydrogen-dominated metallic alloys at more accessible pressure in 2004 [3]. High *T*_c_ in pressurized hydrogen sulfide was theoretically proposed by Yan-ming Ma's group [4] and discovered in high-pressure experiments by Eremets’ group [1]. This Special Topic features two comprehensive reviews, two original research articles and five insightful perspectives, which offer some of the most recent advancements and also different views in this field.

The review paper by Y. Sun *et al.* [5] provides a comprehensive overview of the present status of the progress in the study of clathrate metal superhydrides. The key criteria for high-*T*_c_ superhydrides include the novel clathrate structural motif bonded by hydrogen atoms and the major participation of hydrogen electrons at the Fermi surface. Such theoretical revelations are firmly supported by the experimental realization of the *T*_c_ of 215 K in CaH_6_ at 170 GPa and the discovery of near room-*T*_c_ at 260 K in LaH_10_ [6]. The other review paper by W. Zhao *et al.* [7] summarizes recent progress in ternary hydrides, which have richer chemical compositions and structural prototypes than binary hydrides, and hence may host more novel properties with the possibility of high *T*_c_ at lower pressures. The issues and challenges of ternary hydrides are also presented together with the prospects and opportunities of this class of hydrides. In a research article by K. Lu *et al.* [8], superconductivity in antimony polyhydride is reported for the first time with a high *T*_c_ of 116 K at a pressure of 184 GPa. An upper magnetic field is estimated to be 20 Tesla. The second research article authored by D. Peng *et al.* [9] concerns the nature of the near-room-temperature resistance transition in lutetium with H_2_/N_2_ gas mixture under pressure. With a comprehensive in-depth study, the authors could repeatedly reproduce the near-room-temperature upsurge of electrical resistance in the Lu–H–N system, but the ostensive ‘superconductivity’ is actually a metal-to-semiconductor transition. Note that the astonishing publication of room-*T*_c_ in the Lu–H–N system at merely 1 GPa of pressure [10] was retracted within eight months after dozens of negative reproducibility reports (e.g. [11]).

The five perspectives in this Special Topic cover various views in the field. Mikhail Eremets, a leading pioneer in high-pressure hydrides, emphasizes the importance of robust evidence and the reproducibility of superconductivity [12]. This is because of the formidable challenges in high-pressure experiments for high-*T*_c_ hydrides, including the containment of hydrogen, synthesis of hydrogen-dominant compounds, demonstration of zero resistivity and characterization of the Meissner state, all *in situ* at multi-megabar pressures on micrometer-sized samples through massive pressure vessels. One of us (Ho-Kwang Mao), in his perspective ‘Pressure-induced hydrogen-dominant high-temperature superconductors’ [13], discusses the apparent contradiction between the prerequisite condition of ambient pressure for high-*T*_c_ application and our fundamental understanding that hydrogen-dominant high-*T*_c_ superhydrides require enormous pressure. In a perspective paper by Peng-fei Shan, Liang Ma and Jinguang Cheng [14], ternary superhydrides for high *T*_c_ at low pressures are discussed. The A–B–H_*x*_ ternary systems have already shown promising potentials of higher *T*_c_ and lower pressure, as these authors show, which may shed light on the room-*T*_c_ at ambient pressure. In the fourth perspective paper, Viktor Struzhkin and Ho-Kwang Mao [15] summarize magnetic methods in the study of high-*T*_c_ hydrides in a diamond anvil cell. While the electrical transport measurements in the field are well established, the Meissner state determination for the minute samples in the ultra-high-pressure vessel is significantly harder and has provoked critiques of claims of superconductivity in these new hydride materials. They believe that weak but definitive signals of the Meissner state have been observed at extreme pressures. The room-*T*_c_ rush comes with many caveats. Jorge Hirsch [16], the leading skeptic of superhydride superconductors, focuses on perceived uncertainties and detailed discrepancies in specific measurements. His meticulous statistical analysis of background noise signals [17] has led to the retraction of the first published room-*T*_c_ in the C–S–H system [18].

With the maximum *T*_c_ of LaH_10_ at 260 K and 190 GPa, the pinnacle of room-*T*_c_ superconductivity appears tantalizingly close [12], but the periodic-table-wide search for A in A–H_*x*_ binary systems has already exhausted the explorable pressure–temperature–composition phase space. The frontier has now moved to the next open fields of multicomponent systems. The idea of high-*T*_c_ superhydrides actually originated from the quest for metallic hydrogen [2,13]. The fundamental impasse of the high-*T*_c_ superhydrides is the reliance on the specific bonding form and electronic structure of the hydrogen that can only be achieved at high pressures. Materials application requires the sustention of high-pressure conditions for ambient usage [19].
